# Harnessing advanced large language models in otolaryngology board examinations: an investigation using python and application programming interfaces

**DOI:** 10.1007/s00405-025-09404-x

**Published:** 2025-04-25

**Authors:** Cosima C. Hoch, Paul F. Funk, Orlando Guntinas-Lichius, Gerd Fabian Volk, Jan-Christoffer Lüers, Timon Hussain, Markus Wirth, Benedikt Schmidl, Barbara Wollenberg, Michael Alfertshofer

**Affiliations:** 1 Department of Otolaryngology, Head and Neck Surgery, TUM School of Medicine and Health, Technical University of Munich (TUM), Ismaningerstrasse 22, 81675 Munich, Germany; 2 Department of Otorhinolaryngology, Jena University Hospital, Friedrich-Schiller-University Jena, 07747 Jena, Germany; 3 Department of Otorhinolaryngology, Head and Neck Surgery, Medical Faculty, University of Cologne, 50937 Cologne, Germany; 4 Department of Oral and Maxillofacial Surgery, Institute of Health, Charité– Universitätsmedizin Berlin, Corporate Member of Freie Universität Berlin, Humboldt-Universität zu Berlin, Berlin, 10117 Berlin, Germany

**Keywords:** Large language models, Otolaryngology education, Board examinations, APIs, Medical AI integration, Python programming

## Abstract

**Purpose:**

This study aimed to explore the capabilities of advanced large language models (LLMs), including OpenAI’s GPT-4 variants, Google’s Gemini series, and Anthropic’s Claude series, in addressing highly specialized otolaryngology board examination questions. Additionally, the study included a longitudinal assessment of GPT-3.5 Turbo, which was evaluated using the same set of questions one year ago to identify changes in its performance over time.

**Methods:**

We utilized a question bank comprising 2,576 multiple-choice and single-choice questions from a German online education platform tailored for otolaryngology board certification preparation. The questions were submitted to 11 different LLMs, including GPT-3.5 Turbo, GPT-4 variants, Gemini models, and Claude models, through Application Programming Interfaces (APIs) using Python scripts, facilitating efficient data collection and processing.

**Results:**

GPT-4o demonstrated the highest accuracy among all models, particularly excelling in categories such as allergology and head and neck tumors. While the Claude models showed competitive performance, they generally lagged behind the GPT-4 variants. A comparison of GPT-3.5 Turbo’s performance revealed a significant decline in accuracy over the past year. Newer LLMs displayed varied performance levels, with single-choice questions consistently yielding higher accuracy than multiple-choice questions across all models.

**Conclusion:**

While newer LLMs show strong potential in addressing specialized medical content, the observed decline in GPT-3.5 Turbo’s performance over time underscores the necessity for continuous evaluation. This study highlights the critical need for ongoing optimization and efficient API usage to improve LLMs potential for applications in medical education and certification.

## Introduction

The field of artificial intelligence (AI) has witnessed considerable improvement, leading to the development of systems capable of addressing complex tasks across a range of industries [1]. Among the most notable advancements in AI are machine learning and large language models (LLMs), which have enhanced the capabilities of automated systems to process, comprehend, and generate human language [2]. These models leverage extensive datasets and algorithms to generate human-like responses, demonstrating capabilities previously associated with human cognition [3]. Key players such as OpenAI’s generative pretrained transformer (GPT) series, Google’s Gemini models, and Anthropic’s Claude platforms represent the forefront of this technology and are designed to not only generate text but to do so in a manner that reflects a deep understanding of context, subtlety, and even abstract concepts [4–6].

The implications of these advancements are far-reaching, with applications spanning from customer service automation to creative writing and beyond [7]. In medical education, LLMs have demonstrated the potential to generate educational content, simulate patient interactions, and assess medical knowledge through automated questioning systems [8–10]. However, their application in highly specialized fields like otolaryngology remains underexplored [11]. Otolaryngology encompasses a wide array of subspecialties, including audiology, phoniatrics, rhinology, laryngology, sleep medicine, and head and neck oncology, each requiring a high degree of domain-specific expertise. Evaluating LLMs on specialized medical content, such as otolaryngology board questions, is critical for several reasons. Firstly, it can inform the development of AI tools tailored to assist trainees and professionals in mastering complex topics. Secondly, successful integration of LLMs in such contexts could improve access to high-quality educational resources, especially in regions with limited specialist educators. Finally, understanding the limitations of LLMs in this field helps identify areas where human expertise remains indispensable, thus ensuring patient safety and the reliability of medical practice.

The primary objective of this study was to explore the capabilities of multiple advanced LLMs, including models from OpenAI, Google’s Gemini series, and Anthropic’s Claude series, in addressing highly specialized otolaryngology board examination questions. Additionally, the study included a longitudinal assessment of GPT-3.5 Turbo, which was evaluated using the same set of questions one year ago [12]. This comparison over time is critical for understanding how updates to LLMs—such as changes to their underlying architecture, algorithmic optimizations, or expansions in training datasets—affect their ability to handle specialized medical content. Long-term evaluations provide a unique perspective on the stability and reliability of LLMs for high-stakes applications, helping to identify whether advancements in general capabilities come at the expense of performance in niche domains. Through a methodical approach that utilizes Python programming and interactions with Application Programming Interfaces (APIs), this study pioneers a novel strategy for assessing the efficacy of AI in educational settings within the specialized context of otolaryngology.

## Methods

### Study design

The study design facilitated the evaluation of multiple AI models in ENT using a semi-automated process. Questions from an otolaryngology-specific database were automatically answered by each model and classified as correct or incorrect. This approach mimics the manual process of querying an LLM’s interface, allowing us to efficiently assess the reliability of different LLMs for answering specialty-specific questions.

### Question database

The question bank, curated by Prof. Dr. med. Jan-Christoffer Lüers, mirrors real board exam scenarios and has been validated by experts for accuracy and relevance. It forms the basis of the German version of the ORL App (https://jj-solutions.de/), which uses gamification to enhance knowledge among aspiring ENT professionals through competitive quizzes. Users, who are medical professionals, can submit new questions, which undergo expert verification before being added. The platform is funded by the German Society of Oto-Rhino-Laryngology, Head and Neck Surgery, and it covers a wide array of 15 otolaryngology subspecialties. These include e.g. allergology, audiology, head and neck tumors, face and neck, inner ear and skull base, larynx, middle ear, oral cavity and pharynx, nose and sinuses, phoniatrics, salivary glands, sleep medicine, the vestibular system, and legal aspects. To maintain the study’s integrity, we omitted any questions that relied on images. The study included a total of 2,576 questions, which were divided into multiple-choice (479 questions) and single-choice (2,097 questions). Official permission was secured from the copyright owner before beginning the study to use the questions for research purposes.

### AI models

The response-testing of the different AI models was conducted by one author (P.F.F.) between August 27th and September 4th, 2024, utilizing Python programming language scripts to handle the vast number of 28,336 questions entered to all the AI models by interacting with the APIs. We selected the 11 models based on their overall performance and their comparability to existing data. Since OpenAI released ChatGPT, only Anthropic’s Claude and Google’s Gemini have been comparable and freely accessible. Each series includes versions differing in performance, speed, and cost. Therefore, we included all current versions of Claude and Gemini, along with the main GPT-4 models. GPT-3.5, used on the same database a year ago, was included to assess performance changes over time, despite being an older model. Facebook’s Llama was excluded as it was unavailable in Germany at the study’s time. We utilized the most recent stable versions of the following ChatGPT models: GPT-3.5 Turbo, GPT-4, GPT-4 Turbo, GPT-4o, and GPT-4o mini (https://platform.openai.com/docs/models). We engaged the latest Gemini models from Google: Gemini 1.0 Pro, Gemini 1.5 Flash, and Gemini 1.5 Pro (https://ai.google.dev/gemini-api/docs/models/gemini). Finally, we incorporated the most recent Claude models: Claude 3 Haiku, Claude 3.5 Sonnet, and Claude 3 Opus (https://docs.anthropic.com/en/docs/about-claude/models).

For each model the default API settings were maintained throughout the study, with the exception of the Gemini models. For these, we adjusted the safety settings to block none for all four harm categories (Hate Speech, Harassment, Sexually Explicit, and Dangerous Content). This adjustment was necessary to ensure the models did not withhold responses to medical questions that might be erroneously classified as potentially harmful. It is important to note that, to our knowledge, the APIs for ChatGPT and Claude do not offer the option to modify harm categories.

### API access and cost structure

To access the APIs, platform providers impose a fee structured as a cost per thousand tokens. These tokens, which are the fundamental units used by LLM platforms, help quantify the amount of text processed. A token typically represents a word, punctuation mark, or character, with approximately 750 words corresponding to 1,000 tokens. In our study, we consistently employed the most recent versions of each model available.

The Claude and Gemini APIs are currently accessible free of charge at the lowest rate limits, which range from 2 to 15 requests per minute depending on the model. Notably, the Gemini 1.5 models have tiered pricing, where costs double for prompts exceeding 128 K tokens; however, this scenario did not occur in our study. The paid usage tiers we utilized offered rate limits that enabled us to complete data collection from all 11 LLMs in less than two hours. This efficiency was achieved by employing individual API keys for each model, allowing us to make parallel requests to the respective companies’ servers. To expedite data collection, we incurred usage-based costs, spending approximately $23 on the GPT models and $9 on the Claude models. No costs were incurred for the Gemini models, thanks to a Google-provided three-month trial that included $300 in credits, which covered our higher usage rates. API implementation was straightforward due to comprehensive documentation. Rate limits posed minor challenges: ChatGPT and Claude initially required Python script timers until higher tier access resolved the restrictions. Google’s Gemini, available through a free trial, needed no timing adjustments. Aside from Gemini’s harm categories, no other API challenges were encountered.

### Prompt design for LLM interaction

To facilitate a new analysis step using Python code, we tailored the wording of our prompts to explicitly direct the LLMs to refrain from offering additional explanations. This modification was essential as the selected LLMs often attempt to justify why alternative answers were not chosen, which could have complicated our data extraction process. The prompts underwent refinement through trial runs, designed to elicit responses from LLMs with only the correct answer letter(s) A, B, C, D. Initial prompts were effective for all ChatGPT versions, but only Gemini 1.0-Pro, Claude Opus, and Haiku initially met this criterion among the other models. Adjustments were made to develop a prompt version universally compatible across the models. These prompts facilitated receiving the desired response structure without altering question content, ensuring applicability to all question types used in this study.

Each prompt, including one question, was submitted to the API only once, without modification or translation. To accommodate differences in question formats, we employed two distinct prompts in German when soliciting responses from the corresponding AI model to quiz-style questions with four options. The original German prompts, along with two examples each of multiple-choice and single-choice questions, can be accessed in the Excel sheet or Jupyter notebooks available on GitHub (https://github.com/pafu23/hno-app-database-api-request).

When addressing single-choice questions, the following prompt was utilized:

(A) “Please answer the following question. Only one answer option is correct. Enter only the correct answer letter. Avoid additional explanations or information:

Single choice question.

- Option A.
- Option B.
- Option C.
- Option D”.

We used the following prompt for multiple-choice questions:

(B) “Please answer the following question. Several answer options could be correct. Enter only the correct answer letters. Avoid additional explanations or information:

Multiple choice question.

- Option A.
- Option B.
- Option C.
- Option D”.

### Data collection and processing

Using Anaconda, a Python data science platform (Anaconda, Inc., Austin, Texas, USA), we executed our Python code within a Jupyter notebook. Jupyter notebook is an open-source web application that enables users to create and share documents containing live code, equations, visualizations, and narrative text. We operated this application on a Python 3 kernel, an execution environment that interprets Python code. Our Python scripts (https://github.com/pafu23/hno-app-database-api-request) performed the following tasks sequentially:

i. Initially, the code imports the necessary Python libraries, which are collections of modules that enhance Python’s capabilities without requiring additional code. For example, the library *openpyxl* allows for handling Excel sheets.
ii. The script loads the prepared Excel table. Column B indicates whether the entry is a multiple-choice question; if not, it’s classified as a single-choice question. Column C contains the question itself, while Columns D through G list the possible answers. Columns H to BU document the created prompt, the response received from the LLM, and the analysis of the answer, as detailed in steps v and vi.
iii. We defined a configuration variable (*model_config*) in the script to facilitate switching between different AI sub-models and to specify where data should be stored in the Excel file based on the LLM used.
iv. The Jupyter notebook features a function that sends prompts to the respective AI model’s API and receives responses.
v. In our Python code, we used the *update_response_columns* function along with a regular expression (regex) pattern to identify which letters (A to D) the LLMs selected as correct answers. A regex pattern is a sequence of characters that forms a search pattern, often used for string matching or manipulation. By crafting prompts that elicited responses containing only the correct letters, our code could accurately detect and record these responses as binary values (0 for incorrect, 1 for correct).
vi. The code iterates through rows in the Excel sheet, each representing a question. It determines whether the question is multiple-choice or single-choice, selects the appropriate prompt format, and combines it with the question and answer options. This compiled prompt is then logged in the Excel sheet to facilitate manual validation, sent to the LLM’s API, and the raw API response is also stored for potential review.
vii. Additional functions, such as an API request counter and intermediate data saving, are implemented to prevent errors related to API rate limits and potential data loss. Ultimately, the updated Excel file is saved by overwriting the original file, thereby securing the collected data.

Manual quality control in Excel involved visually checking responses for excess information, feasible due to the four-letter answer limit. Row sums verified that only one answer was given for single-choice questions and that multiple-choice answers were correctly marked, essential for subsequent SPSS analysis. Conditional formatting quickly identified rare instances where LLMs provided no answers.

The performance of each LLM was evaluated based on its accuracy, defined as the proportion of questions answered correctly compared to the reference answers from the online study platform. A higher accuracy corresponds to a greater number of correct answers, serving as the primary performance indicator in this study.

In multiple-choice questions, a response was deemed correct only if the LLM identified all correct options, with no partial credit awarded. For both single and multiple-choice questions, responses were marked incorrect if no answer letter was selected. In these rare instances, the models attempted to justify their inability to choose an answer.

### Statistical analysis

We utilized Pearson’s chi-square test to assess differences in question format and categories. Due to the non-normal distribution of the data, Mann-Whitney-U test was employed for the comparison of single-choice and multiple-choice questions as well as for negated and non-negated questions. To compare the current performance of GPT-3.5 Turbo with previously reported results, we utilized the Wilcoxon signed-rank test, given the non-parametric nature of the data. The statistical analysis was carried out using SPSS Statistics 25 (IBM, Armonk, NY, USA), and a two-tailed p-value of ≤ 0.05 was used to determine statistical significance.

## Results

### Longitudinal comparison of GPT-3.5 turbo

A comparison of current results with those previously reported by our group, using the same set of questions, revealed a statistically significant decline in performance for GPT-3.5 Turbo [12]. The accuracy decreased from 57.3 to 52.6%, with a significance level of *p* < 0.001. A statistically significant difference in the performance of the model was found in the following question categories with current vs. previous performance, respectively: Oral cavity & pharynx (55.1% vs. 33.1%; *p* < 0.001), Allergology (62.2% vs. 72.2%; *p* = 0.003), Larynx (49.2% vs. 59.7%; *p* = 0.028), Face & neck (61.2% vs. 71.9%; *p* = 0.002), Head and neck tumors (56.8% vs. 65.0%; *p* = 0.026), Inner ear & skull base (46.4% vs. 56.4%; *p* = 0.009), Vestibular system (52.0% vs. 62.5%; *p* = 0.049), and Sleep medicine (52.1% vs. 64.8%; *p* = 0.039).

### Overall LLM performance

**Fig. 1:**
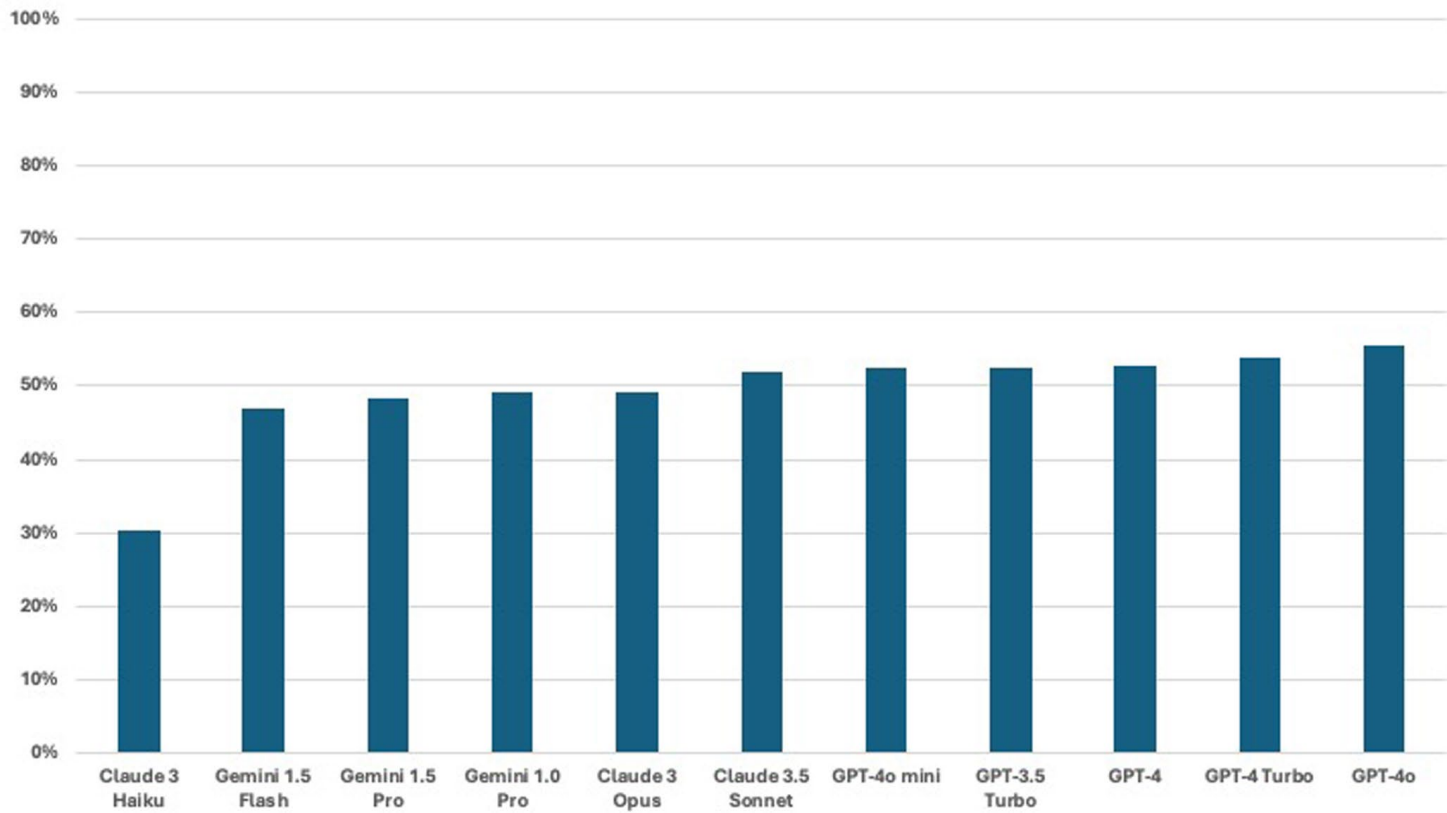
Bar graph illustrating the overall accuracy of the different large language models (LLMs) investigated in this study. Performance is measured as the percentage of correctly answered otolaryngology board examination questions out of a total of 2,576 questions. The models compared include Claude 3 Haiku, Gemini 1.5 Flash, Gemini 1.5 Pro, Gemini 1.0 Pro, Claude 3 Opus, Claude 3.5 Sonnet, GPT-4o mini, GPT-3.5 Turbo, GPT-4, GPT-4 Turbo, and GPT-4o. Higher accuracy rates indicate better performance, with GPT-4o achieving the highest accuracy and Claude 3 Haiku the lowest

The overall performance of the investigated LLM-chatbots, assessed in terms of accuracy (the proportion of correctly answered questions out of 2,576), was as follows: Gemini 1.5 Flash achieved 46.8% (*n* = 1,205), Gemini 1.5 Pro 48.3% (*n* = 1,243), and Gemini 1.0 Pro 49.1% (*n* = 1,266). Claude 3.5 Sonnet scored 51.9% (*n* = 1,337), Claude 3 Opus 49.1% (*n* = 1,264), and Claude 3 Haiku 30.2% (*n* = 779). GPT-3.5 Turbo reached 52.6% (*n* = 1,354), GPT-4 52.8% (*n* = 1,361), GPT-4o mini 52.5% (*n* = 1,352), GPT-4 Turbo 54.0% (*n* = 1,391), and GPT-4o 55.6% (*n* = 1,431). Figure 1 depicts the overall performance of the different LLMs that were examined in this study.

### Performance of single-choice versus multiple-choice questions

**Fig. 2:**
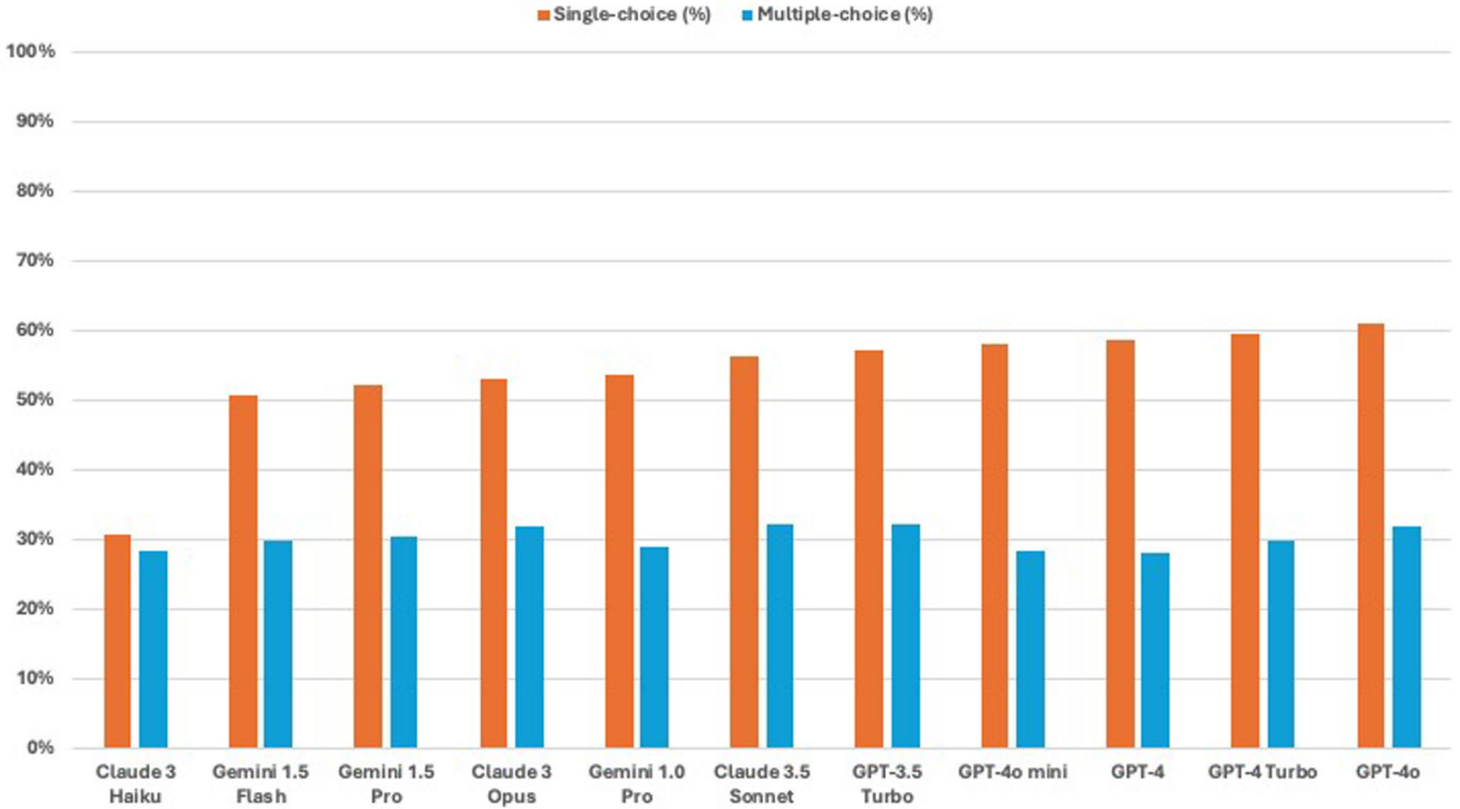
Paired bar graph comparing the performance of different large language models (LLMs) on single-choice and multiple-choice otolaryngology board examination questions. Performance is measured as the percentage of correctly answered questions out of a total of 2,576 questions, with single-choice question accuracy represented in orange and multiple-choice question accuracy in blue. Models evaluated include Claude 3 Haiku, Gemini 1.5 Flash, Gemini 1.5 Pro, Gemini 1.0 Pro, Claude 3 Opus, Claude 3.5 Sonnet, GPT-4o mini, GPT-3.5 Turbo, GPT-4, GPT-4 Turbo, and GPT-4o. Across all models, accuracy on single-choice questions consistently exceeds that on multiple-choice questions, highlighting a common challenge for LLMs in addressing more complex question formats. GPT-4o demonstrates the highest accuracy for both question types, while Claude 3 Haiku performs the lowest overall

Across all LLMs tested, performance on single-choice questions consistently outperformed that on multiple-choice questions. The results were as follows: The Gemini 1.5 Flash model scored 50.7% on single-choice versus 29.9% on multiple-choice (*p* < 0.001), Gemini 1.5 Pro achieved 52.3% against 30.5% (*p* < 0.001), and Gemini 1.0 Pro recorded 53.7% versus 29.0% (*p* < 0.001). For the Claude series, the Claude 3.5 Sonnet scored 56.4% on single-choice versus 32.2% on multiple-choice (*p* < 0.001), Claude 3 Opus achieved 53.0% against 31.9% (*p* < 0.001), while Claude 3 Haiku had 30.6% versus 28.4% (*p* = 0.199), indicating no significant difference. The GPT-3.5 Turbo model recorded 57.2% on single-choice compared to 32.2% on multiple-choice (*p* < 0.001). The scores for GPT-4 were 58.5% and 28.0% (*p* < 0.001), for GPT-4o mini were 58.1% and 28.2% (*p* < 0.001), for GPT-4 Turbo were 59.5% and 29.9% (*p* < 0.001), and for GPT-4o were 61.0% and 31.9% (*p* < 0.001). Figure 2 visualizes the performance of distinct LLMs for single-choice and multiple-choice questions.

### Performance of negated versus non-negated questions

In examining the performance differential between negated and non-negated questions for each LLM, varied results were observed. Notably, all versions of GPT-4 displayed improved performance on negated questions compared to their non-negated counterparts, with statistically significant improvements noted in three instances: GPT-4 (58.4% vs. 51.5%, *p* = 0.005), GPT-4o mini (56.4% vs. 51.5%, *p* = 0.048), and GPT-4o (60.0% vs. 54.5%, *p* = 0.026). For the GPT-3.5 Turbo model, there was a small, non-significant performance improvement noted on negated questions (54.6% vs. 52.1%, *p* = 0.297). The GPT-4 Turbo model similarly showed little to no performance difference (55.0% vs. 53.7%, *p* = 0.603). For the Gemini models, the performance was similar between non-negated and negated questions, though none reached statistical significance: Gemini 1.5 Flash (45.9% vs. 50.5%, *p* = 0.061), Gemini 1.5 Pro (47.7% vs. 50.5%, *p* = 0.260), and Gemini 1.0 Pro (48.8% vs. 50.5%, *p* = 0.498). Claude models presented mixed results. Claude 3.5 Sonnet and Claude 3 Opus demonstrated a minor difference in performance with no significant difference (52.1% vs. 51.3%, *p* = 0.755 and 49.7% vs. 46.4%, *p* = 0.172, respectively). Conversely, Claude 3 Haiku showed a significant decrease in performance for negated questions (32.6% vs. 20.5%, *p* < 0.001).

### Performance by question length

Statistically significant influences of question length on the performance were found for all models with Gemini 1.5 (rp = -0.109 and *p* < 0.001), Gemini 1.5 Pro (rp= -0.152 and *p* < 0.001), Gemini 1.0 Pro (rp= -0.136 and *p* < 0.001), Claude Sonnet 3.5 (rp= -0.093 and *p* < 0.001), Claude Opus 3.0 (rp=-0.105 and *p* < 0.001), Claude Haiku 3.0 (rp=-0.060 and *p* = 0.002), ChatGPT 3.5 (rp=-0.043 and *p* = 0.029), ChatGPT 4 (rp = -0.133 and *p* < 0.001), ChatGPT 4o mini (rp=-0.085, *p* < 0.001) and ChatGPT 4 turbo (rp=-0.121, *p* < 0.001).

### Performance by otolaryngology subspecialty

GPT-4o demonstrated superior accuracy in several categories, particularly in allergology (66.5%), head and neck tumors (62.8%), and the vestibular system (61.2%). It also showed strong performance in sleep medicine (59.2%) and the face & neck category (65.7%). Similarly, GPT-4 Turbo excelled, especially in the face & neck category, where it achieved the highest accuracy among all LLMs at 66.5%. It also performed well in phoniatrics (59.8%) and salivary glands (54.3%), along with a solid showing in the inner ear & skull base (53.6%) and head and neck tumors (60.7%). GPT-4 consistently ranked among the top performers, leading all models in allergology with an accuracy of 69.9%. It also achieved strong results in the oral cavity & pharynx (60.6%), face & neck (60.3%), salivary glands (54.3%), and the vestibular system (58.6%). However, the category of facts & history posed challenges across all LLMs, with GPT-4o reaching the highest accuracy at 44.4%, which was notably lower than its performance in other categories. Legal aspects also proved difficult, with GPT-4 Turbo achieving the highest accuracy in this category at 38.6%. Claude 3 Haiku consistently exhibited lower performance across most categories, frequently achieving accuracies below 40%. The performance of the LLMs across various otolaryngology question categories is summarized in Table 1.

**Table 1:**
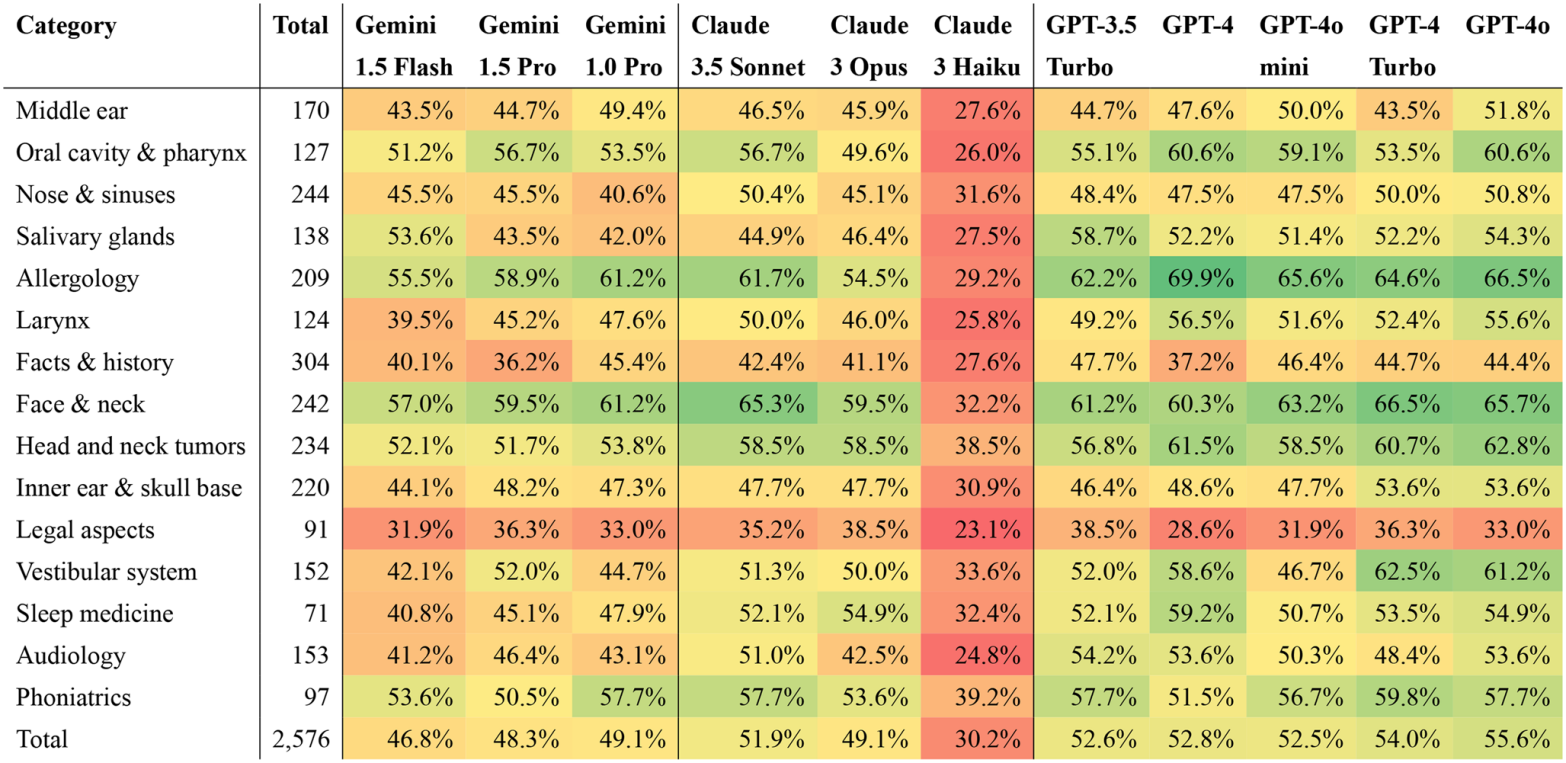
Performance of various large Language models (LLMs), stratified by otolaryngology question category. The table displays the accuracy (%) of each model in answering otolaryngology board examination questions across 15 categories, including “middle ear,” “oral cavity & pharynx,” “head and neck tumors,” and others. Each category’s total number of questions is provided to indicate the weight of each domain. Models evaluated include the gemini 1.5 flash, gemini 1.5 pro, gemini 1.0 pro, Claude 3.5 sonnet, Claude 3 opus, Claude 3 Haiku, GPT-3.5 turbo, GPT-4, GPT-4 turbo, GPT-4o Mini, and GPT-4o. GPT-4o achieved the highest overall accuracy, excelling particularly in allergology (66.5%), face and neck (65.7%), and head and neck tumors (62.8%). Claude 3 Haiku showed the lowest overall accuracy, with notable underperformance in several categories

## Discussion

The findings of this study provide a comprehensive evaluation of the performance of various LLMs in addressing the specialized domain of otolaryngology, particularly within the context of board examination preparation. This study is particularly valuable as it not only assesses the capabilities of the latest models from OpenAI, Google, and Anthropic but also revisits the performance of GPT-3.5 Turbo, offering insights into the progress (or regression) of these models over time.

The results of this study reveal a varied performance landscape among the tested LLMs, with newer or more advanced models, such as GPT-4 and its variants, consistently outperforming their predecessors, including GPT-3.5 Turbo. This trend aligns with findings from previous research demonstrating the superior capabilities of GPT-4 in educational otolaryngology applications. For instance, studies evaluating GPT-4’s performance in otolaryngology-specific tasks showed that GPT-4 achieved an accuracy of 86% on rhinology standardized board questions, significantly surpassing GPT-3.5, which only scored 45.2% [13]. Similarly, GPT-4 achieved a passing rate of 75% on open-ended Canadian Otolaryngology board examination questions, emphasizing its capability in handling nuanced clinical scenarios [14]. Moreover, GPT-4 demonstrated strong proficiency in otolaryngology-specific clinical knowledge, correctly answering 77.1% of 4,566 multiple-choice questions, outperforming other LLMs like MedPaLM and GPT-3.5 [15]. Despite these promising results, our findings indicate that GPT-4o and similar models achieved only 50–60% accuracy across otolaryngology board questions. This disparity with prior studies may be attributed to several factors. First, the language of the questions (German in this study) presents an additional complexity for LLMs, especially if they were primarily trained on English-language datasets. Translation or linguistic nuances in German medical terminology may have contributed to errors in comprehension or reasoning. Second, the question design used in this study—derived from a German otolaryngology board preparation platform—differs from standardized board exams in other countries. Questions requiring knowledge of region-specific guidelines or clinical practices may not align with the LLM’s training data. Third, the contextual depth and formatting of questions, particularly multiple-choice items, likely impacted performance. It has been well-documented that LLMs are sensitive to the structure and wording of multiple-choice questions [12, 16]. Questions that present subtle distinctions between answer options or rely on higher-order reasoning can disproportionately challenge models that prioritize pattern recognition over true conceptual understanding. Fourth, training and fine-tuning variations across different studies may lead to performance disparities. For example, prior studies may have included domain-specific tuning of LLMs, whereas our evaluation used the general-purpose versions of the models.

The performance of the Claude models, particularly Claude 3.5 Sonnet and Claude 3 Opus, highlights their potential competitiveness with the GPT series, although they generally fall slightly behind in overall accuracy. Claude 3.5 Sonnet, with an accuracy of 51.9%, and Claude 3 Opus, at 49.1%, demonstrate a robust ability to handle specialized tasks. However, they have not yet reached the top-tier performance levels seen in the GPT-4 variants. Interestingly, a recent study that evaluated the diagnostic performance of GPT-4o and Claude 3 Opus using radiology quiz cases found that Claude 3 Opus outperformed GPT-4o, underscoring that performance can vary significantly depending on the specific domain and task at hand [17]. Similarly, Schmidl et al. demonstrated that Claude 3 Opus surpassed GPT-4 in providing accurate information on the diagnostic workup and treatment of patients with primary head and neck squamous cell carcinoma [5]. The significantly lower accuracy of Claude 3 Haiku, at 30.2%, further suggests that not all versions within a model series are equally optimized for complex tasks, indicating variability in performance based on specific model iterations or intended use cases.

The Gemini models generally lag behind both the GPT and Claude models in overall accuracy. The top-performing Gemini model, Gemini 1.0 Pro, achieved an accuracy of 49.1%, which is comparable to Claude 3 Opus but still falls short of the performance exhibited by the GPT variants. A study assessing the diagnostic capabilities of GPT-4o, Claude 3 Opus, and Gemini 1.5 Pro using radiology quiz cases found that Claude 3 Opus outperformed both GPT-4o and Gemini 1.5 Pro [17]. This aligns with our study’s findings, where Gemini 1.5 Pro also demonstrated lower accuracy compared to Claude 3 Opus, reinforcing the observation that while the Gemini models are capable, they tend to underperform relative to their Claude counterparts in specialized tasks.

The decline in GPT-3.5 Turbo’s performance on otolaryngology board examination questions, with accuracy dropping significantly from 57.3 to 52.6% (*p* < 0.001), is a noteworthy finding [12]. With the exception of the Oral cavity & pharynx category, all other categories exhibited a decline in performance. This deviation from the overall trend may be attributed to the relatively smaller sample size of 127 questions in this category, making it more susceptible to skew. One plausible explanation for this decrease could be changes in the underlying architecture or algorithms of GPT-3.5 Turbo [18]. As OpenAI regularly updates its models, modifications intended to enhance general performance, improve ethical alignment, or strengthen safety protocols might unintentionally impact the model’s ability to handle highly specialized tasks, such as answering detailed medical exam questions. Another important factor could be updates or expansions in the training data over the past year [19]. While broader and more diverse datasets generally increase a model’s versatility, they can also introduce noise or reduce the model’s focus on specific domains. If the newly incorporated data is more generalized and less specialized, this might have led to a loss of precision in the model’s ability to accurately address niche areas such as otolaryngology. Additionally, the slight modification in prompt design for this year’s analysis may have contributed to the observed performance decline [20]. While the previous prompt was incompatible with the Python script used in this study, the new prompt might have inadvertently altered how GPT-3.5 Turbo interprets and responds to the questions.

Another critical observation from our study is the consistent underperformance of LLMs on multiple-choice questions compared to single-choice questions, a trend that was statistically significant across all models (*p* < 0.001). This aligns with findings from our previous work, as well as other studies, which have shown that LLMs are highly sensitive to the order of options in multiple-choice questions, with performance varying —by as much as 13–75%—depending on the sequence of answer choices [12, 21]. Furthermore, despite ongoing advancements in model design, LLMs continue to exhibit biases, such as a propensity to select certain options like “Option A,” which further undermines their accuracy in multiple-choice contexts [22]. Even models such as GPT-4, which outperform their predecessors in many areas, still struggle with the higher-order reasoning required for complex multiple-choice questions [16]. These questions are often crafted to test deep understanding and the ability to differentiate between closely related concepts, making them particularly challenging for LLMs that may rely more on pattern recognition than true comprehension. In contrast, single-choice formats, which typically present clearer distinctions between correct and incorrect answers, pose fewer difficulties for LLMs. This pattern of underperformance suggests that while LLMs are becoming increasingly adept at handling general knowledge tasks, they continue to face significant challenges in scenarios that require nuanced understanding and advanced reasoning skills.

The analysis of LLM performance on negated versus non-negated questions revealed mixed results across the models. Negated questions, which often involve more complex linguistic structures, present a significant challenge as they require the model to correctly interpret and reverse the meaning of statements, a task that goes beyond simple pattern recognition. Notably, the GPT-4 models demonstrated statistically significant improvements on negated questions compared to non-negated ones. This suggests that the GPT-4 series may have been specifically fine-tuned or inherently better equipped to process and correctly interpret negated constructs, indicating an advanced level of comprehension that is essential for tackling more sophisticated language tasks. However, the poor performance of Claude 3 Haiku on negated questions highlights that not all LLMs have equally benefited from advancements in linguistic processing.

The variations in performance among the different otolaryngology categories highlight specific challenges and advantages related to the structure and nature of the questions themselves, as well as the inherent capabilities and limitations of the LLMs. For instance, the strong performance of GPT-4o in categories such as allergology (66.5%), head and neck tumors (62.8%), and the vestibular system (61.2%) may be attributed to the more structured and fact-based nature of the questions in these areas. Categories like allergology and head and neck tumors often involve questions that rely on well-defined protocols, standardized treatment guidelines, and diagnostic criteria, which LLMs can more easily retrieve and process. Additionally, these fields are more likely to be covered extensively in the diverse datasets used to train these models, allowing them to draw from factual and straightforward information. On the other hand, the face & neck category, where GPT-4 Turbo excelled with an accuracy of 66.5%, may include questions that are more contextually grounded, requiring the model to understand and synthesize detailed clinical scenarios. The success in this category could be due to the model’s ability to handle questions that integrate multiple pieces of information, such as patient history, symptomatology, and treatment outcomes, which are commonly presented in structured and logical sequences within the training data. Conversely, the significantly lower performance across all models in categories like facts & history, where GPT-4o achieved a modest 44.4%, suggests that these questions might involve more abstract or less standardized information. Historical and fact-based questions often require contextual understanding and may involve nuanced distinctions between similar events or concepts, making them harder for LLMs to parse accurately. Unlike clinical guidelines or textbook knowledge, which follow clear patterns and standardized phrasing, historical information is often fragmented, context-dependent, and requires synthesis across disparate sources. LLMs, being optimized for pattern recognition and probabilistic text generation, struggle with historical reasoning due to the need for chronological coherence and deeper contextual interpretation, which are difficult to capture using training data that lacks explicit historical narratives. The challenge here could also stem from the less frequent and less systematic representation of historical medical information in the datasets, which might lead to inconsistencies in the models’ responses. The poor performance in the legal aspects category, where GPT-4 Turbo’s best result was 38.6%, likely reflects the inherent complexity and variability of legal questions. Legal considerations in medicine are often context-specific, involving intricate regulations that can vary significantly between jurisdictions [23]. A major limitation for LLMs in this domain is that legal frameworks require not only recall of factual legal principles but also their logical application to highly specific case-based contexts. The absence of structured legal reasoning pathways in LLMs, along with the probabilistic nature of their responses, makes it difficult for them to apply legal knowledge deterministically. Furthermore, legal data is often scarce, jurisdiction-dependent, and not as extensively represented in publicly available training corpora, limiting the models’ ability to provide accurate, context-aware responses in this domain. LLMs may struggle with these questions due to the scarcity of consistent, high-quality legal data in their training sets, combined with the need to apply general principles to highly specific legal scenarios. Claude 3 Haiku’s overall lower performance across most categories, frequently scoring below 40%, suggests that this model may struggle with the same complexities and nuances that challenge other models, but to a greater degree. Its lower accuracy might reflect a combination of less effective parsing of complex, multi-step questions and a possible focus on broader or more generalized knowledge in its training, which may not align well with the specialized content of otolaryngology exams.

While these findings are specific to otolaryngology, the observed performance trends likely extend to other medical specialties. Similar studies evaluating LLMs in fields such as internal medicine and radiology have reported comparable patterns, where performance is strongest in domains with well-structured, factual content and weakest in areas requiring abstract reasoning, legal interpretation, or historical knowledge [24, 25]. This suggests that the challenges faced by LLMs in otolaryngology are not unique but rather reflect fundamental limitations in their ability to handle complex, less standardized information across medical disciplines. However, specialty-specific variations may still exist, influenced by differences in question complexity, dataset representation, and the availability of structured guidelines within each field. Comparing LLM performance across multiple medical specialties could help clarify the degree to which these trends hold universally.

The findings of this study highlight both educational and clinical implications for the use of LLMs in specialized medical domains. While the relatively low accuracy rates of 50–60% indicate that LLMs, in their current form, are not suitable as standalone tools for high-stakes applications like board examination preparation or clinical decision-making, they hold considerable promise as supplementary resources. In medical education, models like GPT-4o could be employed to simulate board-style questions or provide immediate feedback, fostering independent learning and reinforcing knowledge acquisition. Moreover, by analyzing incorrect responses, LLMs could help trainees identify and address specific knowledge gaps, enabling targeted remediation. From a clinical perspective, LLMs have the potential to serve as auxiliary tools for decision support, particularly in areas where they demonstrated relatively higher accuracy, such as allergology and head and neck tumors. These models could assist clinicians by providing quick access to standardized guidelines, diagnostic criteria, or treatment protocols, thereby streamlining the retrieval of critical information during routine practice.

Future research should focus on fine-tuning LLMs with domain-specific datasets that include standardized guidelines, region-specific protocols, and specialty-focused cases to improve their precision in handling specialized content. Enhancing multilingual training with diverse datasets is also critical to address the challenges posed by non-English medical terminology and improve performance in international contexts. Moreover, incorporating additional performance metrics—such as the quality of explanations, ability to handle ambiguous reasoning, and responsiveness to user feedback—will provide a more comprehensive understanding of LLM utility. Expanding evaluations to include other medical specialties, such as cardiology or neurology, could further explore the generalizability of LLMs. Finally, longitudinal studies tracking model performance over successive updates will offer insights into their stability and adaptability for high-stakes applications.

## Limitations

Despite the comprehensive evaluation presented in this study, several limitations should be acknowledged. First, the reliance on a single set of board examination questions in German may limit the generalizability of our findings. Although this question bank is robust and covers a wide array of otolaryngology subspecialties, its design reflects region-specific clinical practices and educational standards, which may introduce biases. For example, the phrasing of questions, inclusion of regional guidelines, or emphasis on certain subspecialties may influence the performance of LLMs differently than questions from international or multidisciplinary contexts. Consequently, the findings may not fully represent the LLMs’ capabilities across broader medical fields or when applied to other specialties with different knowledge requirements or question styles.

Furthermore, the exclusive use of the German language raises concerns about the transferability of our results to other linguistic contexts. LLMs are trained on multilingual corpora, but their proficiency varies across languages due to differences in data availability, linguistic complexity, and syntactic structure. Certain languages, including English, tend to be overrepresented in training datasets, leading to potential disparities in model performance when handling medical content in less prevalent languages like German. Additionally, some LLMs may process medical terminology differently in German than in English, which could affect accuracy, particularly in cases involving compound words, abbreviations, or context-specific meanings. Given these challenges, future studies should incorporate multilingual question sets to assess whether LLM performance is consistent across languages or if linguistic biases impact medical reasoning and response accuracy.

Second, the study’s design involved a one-time submission of questions to each LLM, which does not account for potential variations in performance that could arise from repeated queries or slight modifications to prompts. While this approach ensured consistency in data collection, it may overlook the models’ ability to learn or adapt over multiple interactions. Additionally, the decision to use default API settings, with the exception of adjusted safety settings for the Gemini models, means that the results might differ if alternative configurations were employed, particularly if these settings could be fine-tuned to better suit the specialized nature of the questions.

Another limitation lies in the inherent variability of LLM updates. The models evaluated in this study, particularly those from OpenAI, Google, and Anthropic, are frequently updated with new training data and algorithmic adjustments. These updates, while aimed at improving overall performance, may introduce changes that affect the models’ abilities to handle specific, niche domains like otolaryngology.

Additionally, the study’s focus on accuracy as the primary metric of performance may not fully capture the nuanced capabilities of these models. While accuracy is a critical factor, other aspects such as the quality of explanations provided, the ability to handle ambiguous or complex questions, and the models’ responsiveness to user feedback were not assessed in this study. These factors are particularly relevant in educational contexts, where the ability to facilitate learning through detailed, accurate explanations is as important as providing the correct answers. Additionally, an important aspect of model performance is the ability to refrain from providing an answer when uncertain, particularly in the medical field, where critical decisions are made. This self-assessment can prevent reliance on vague responses. However, such instances were rare, and since the primary focus of the study was correctly provided answers, these instances were marked as incorrect. It would be valuable for future studies to investigate this behavior further by examining when and how LLMs choose to refrain from providing an answer.

Finally, the question set’s focus on otolaryngology-specific content inherently limits its applicability to other medical specializations. While the findings provide valuable insights into LLM performance in otolaryngology, they do not account for the broader diversity of medical knowledge or the unique challenges posed by other specialties. For example, domains such as cardiology or neurology, which may have different linguistic demands or require more intricate reasoning, could yield varying results. Future research should explore the performance of LLMs using datasets from multiple specialties to better understand their applicability across the medical field.

## Conclusion

This study provides a comprehensive evaluation of advanced LLMs in addressing the specialized requirements of otolaryngology board examinations, offering critical insights into their current capabilities and limitations. Our findings reveal that while the GPT-4 series demonstrated superior performance compared to earlier models, including GPT-3.5 Turbo, its accuracy remains insufficient for standalone use in high-stakes applications such as board examination preparation or clinical decision-making. Notably, this study uniquely highlights the performance variability of LLMs when applied to German-language otolaryngology board questions, revealing significant challenges posed by linguistic and contextual nuances. Additionally, the observed decline in GPT-3.5 Turbo’s accuracy over time underscores the need for longitudinal assessments to monitor the impact of updates on specialized knowledge domains. The performance of Claude and Gemini models, although competitive, further underscores the variability in effectiveness across different LLM platforms. Despite these limitations, LLMs show considerable promise as supplementary tools in medical education, particularly for enhancing independent learning, simulating board examination scenarios, and identifying knowledge gaps. However, their potential can only be fully realized through targeted fine-tuning with domain-specific datasets, improved multilingual capabilities, and rigorous testing in diverse medical contexts. Future advancements in LLM technology must also address their current challenges, such as inconsistencies in accuracy and reasoning, to ensure their safe and effective integration into medical training and practice. By bridging gaps in accessibility to specialized knowledge and streamlining educational processes, LLMs have the potential to revolutionize medical education and support clinicians in their decision-making processes. However, achieving this vision will require a balanced approach that harnesses their strengths while addressing their limitations through ongoing innovation and oversight.
